# Supplementary figures and images for: Partial revision of the neustonic genus Scapholeberis Schoedler, 1858 (Crustacea: Cladocera): decoding of the barcoding results
Source: PeerJ. 2020 Nov 25;8:e10410. doi: 10.7717/peerj.10410 (PMC7698698; doi:10.7717/peerj.10410)

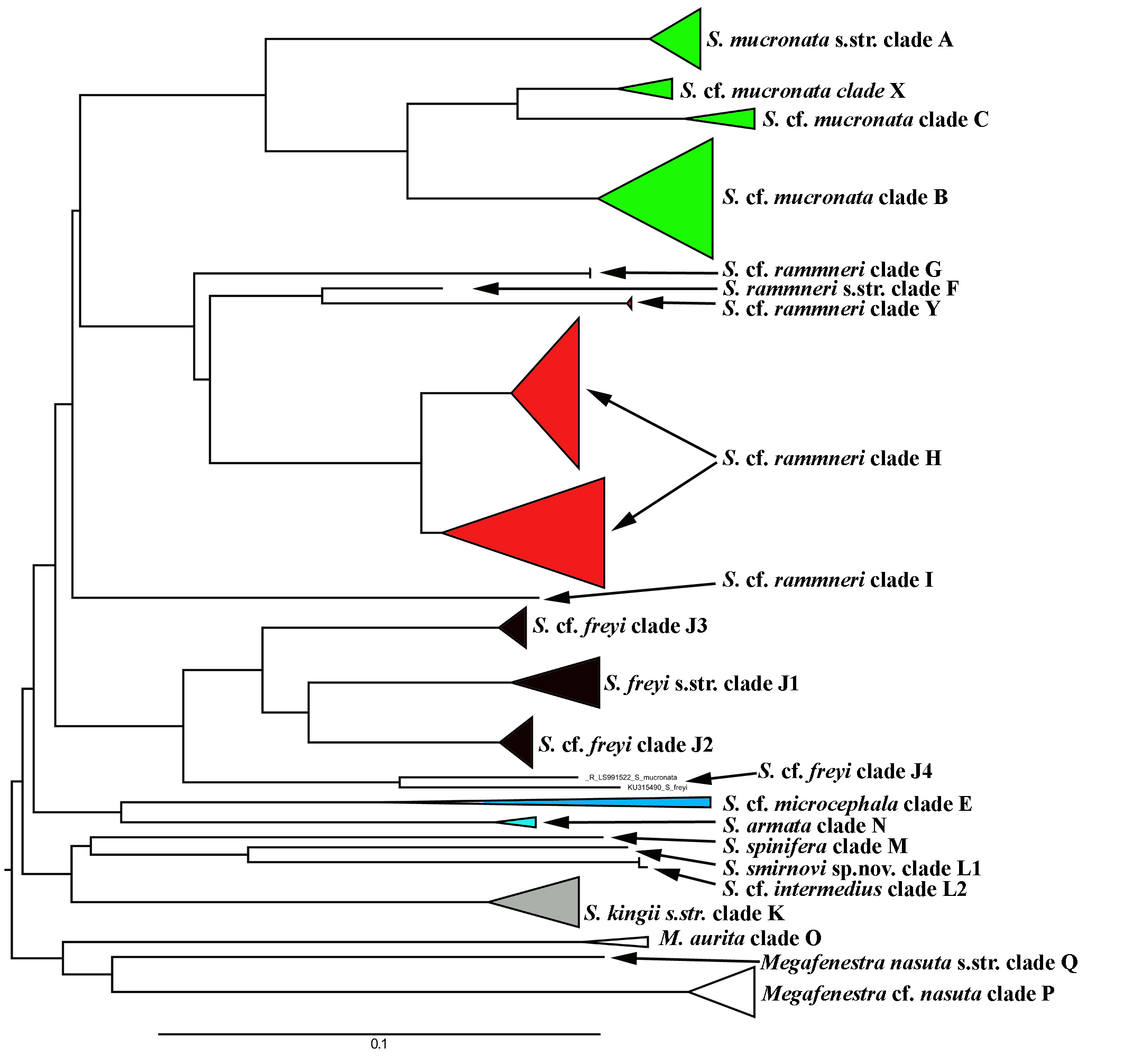

Supplement: Supplemental Information 3 — Bold letters (A–Q, X–Y) indicate geographic clades. Colours represent major species groups in the Scapholeberinae: Scapholeberis mucronata group (green), S. rammneri group (red), S. freyi group (black), S. kingii group (grey), genus Megafenestra (white). The tree is midpoint rooted supporting a basal position of the genus Megafenestra. See Appendix S1 for individual sequences. [file peerj-08-10410-s003.jpg]
